# Modulation of metabolic syndrome components by oral semaglutide in hypothyroid–T2DM patients: a retrospective analysis

**DOI:** 10.25122/jml-2025-0144

**Published:** 2025-12

**Authors:** Dana-Mihaela Tilici, Ruxandra-Mihaela Costinescu, Diana-Loreta Paun, Sorin Constantin Paun, Cristian Guja

**Affiliations:** 1 Doctoral School, Carol Davila University of Medicine and Pharmacy, Bucharest, Romania; 2 University Emergency Hospital of Bucharest, Bucharest, Romania; 3 Carol Davila University of Medicine and Pharmacy, Bucharest, Romania; 4 Bucharest Emergency Clinical Hospital, Bucharest, Romania

**Keywords:** metabolic syndrome, hypothyroidism, type 2 diabetes mellitus, oral semaglutide, GLP-1 receptor agonist, insulin resistance, dyslipidemia, MetS, metabolic syndrome, HDL, high-density lipoprotein, LDL, low-density lipoprotein, T2DM, type 2 diabetes mellitus, IR, insulin resistance, GLP-1, glucagon-like peptide-1, BMI, body mass index, HbA1c, glycated hemoglobin, TSH, thyroid-stimulating hormone, SAH, systemic arterial hypertension, SCH, subclinical hypothyroidism

## Abstract

Metabolic syndrome (MetS) represents the concurrent manifestation of multiple cardiometabolic risk factors, including visceral obesity, hyperglycemia, hypertension, hypertriglyceridemia, and low HDL-cholesterol, cumulatively predisposing to accelerated atherosclerosis and type 2 diabetes mellitus (T2DM). Hypothyroidism frequently coexists with T2DM and further exacerbates insulin resistance (IR), lipid abnormalities, and systemic inflammation, increasing the prevalence and severity of MetS in this population. Oral semaglutide is a glucagon-like peptide-1 receptor agonist approved for T2DM management; however, its impact on MetS parameters in patients with coexisting hypothyroidism remains insufficiently explored. This study aimed to evaluate the effects of oral semaglutide on key MetS components in this high-risk subgroup. We conducted a single-center retrospective cohort study involving 51 adult patients with confirmed hypothyroidism and T2DM, on oral semaglutide (final dose = 14 mg daily) and monitored for 6 months. Clinical and biochemical parameters were analyzed, including glycated hemoglobin (HbA1c), body mass index (BMI), blood pressure, and lipid profile. At 6 months, mean HbA1c decreased by 6.7% (*P* < 0.001), BMI was reduced by 4.04% (*P* < 0.001), triglycerides decreased by 6.7% (*P* < 0.001), and HDL-C increased by 9% (*P* = 0.002). In this observational study, treatment with oral semaglutide was associated with improvements in several components of MetS among patients with coexisting hypothyroidism and T2DM. While these findings suggest a potential therapeutic role for semaglutide in complex metabolic profiles, they should be interpreted with caution due to the study’s design limitations. Further prospective studies are warranted to confirm these observations and to explore the interaction between semaglutide and levothyroxine.

## INTRODUCTION

Metabolic syndrome (MetS) represents a cluster of interconnected cardiometabolic risk factors that significantly increase the likelihood of developing type 2 diabetes mellitus (T2DM), atherosclerotic cardiovascular disease (ASCVD), and all-cause mortality. According to the harmonized definition proposed by the International Diabetes Federation (IDF), the American Heart Association (AHA), and the National Heart, Lung, and Blood Institute (NHLBI), MetS is typically defined by the presence of at least three of the following criteria: central (visceral/abdominal) obesity, hypertriglyceridemia (≥150 mg/dL), decreased serum high-density lipoprotein cholesterol (HDL-C <40 mg/dL in men, <50 mg/dL in women), elevated fasting plasma glucose (≥100 mg/dL), and systemic arterial hypertension (≥130/85 mmHg) [1-3]. It currently affects an estimated 40–46 % of adults worldwide and more than triples the risk of cardiovascular disease and T2DM [4-6].

Emerging evidence underscores the intricate bidirectional relationship between MetS and thyroid dysfunction, particularly hypothyroidism. Both overt and subclinical hypothyroidism have been associated with insulin resistance, dyslipidemia, and visceral adiposity—core components of MetS. Hypothyroidism is a recognized comorbidity in T2DM, with prevalence estimates ranging from ~5 % to 17,5 % [7,8]. Meta-analyses quantify that individuals with subclinical hypothyroidism have 1.28-fold increased odds of MetS, independent of age or sex. Additionally, hypothyroid patients exhibit insulin resistance due to impaired glucose transporter type 4 (GLUT4) expression, decreased hepatic and peripheral glucose disposal, and prolonged insulin half-life [9,10].

The interplay between thyroid function and T2DM is multifaceted and bidirectional [7,8,11-15]. T2DM can influence thyroid hormone metabolism, in part through altered deiodinase activity, while thyroid dysfunction may contribute to dysglycemia, dyslipidemia, elevated blood pressure, and other metabolic imbalances. Individuals with both hypothyroidism and T2DM often present with overlapping metabolic challenges, which may amplify overall cardiometabolic burden [16]. This observational study aimed to describe the metabolic profile of this population and explore its association with oral semaglutide treatment. Because there was no control group, findings are exploratory and hypothesis-generating, not causal.

Dyslipidemia, a hallmark of both MetS and hypothyroidism, is characterized by elevated serum triglycerides and low high-density lipoprotein cholesterol (HDL-C), and may also include increased low-density lipoprotein cholesterol (LDL-C). Thyroid hormones regulate multiple aspects of lipid homeostasis, and their deficiency leads to reduced LDL receptor activity and altered apolipoprotein expression, thereby promoting atherogenesis [17,18]. Furthermore, the association between thyroid dysfunction and dyslipidemia is often underdiagnosed, emphasizing the need for sensitive biomarkers for early detection and risk stratification [11, 17, 19].

Patients with coexisting T2DM and hypothyroidism present a distinct metabolic phenotype, often manifesting a higher burden of MetS components and a poorer glycemic and lipid profile. A promising therapeutic approach has emerged with the development of glucagon-like peptide-1 receptor agonists (GLP-1 RAs). In populations with T2DM, GLP-1 receptor agonists such as oral semaglutide have demonstrated notable reductions in body weight and glycated hemoglobin (HbA₁c), with additional potential benefits for blood pressure and lipid parameters [20-23].

While GLP-1 RAs have demonstrated efficacy in T2DM and obesity, their modulatory effects on MetS components in hypothyroid patients remain insufficiently explored.

**Figure 1:**
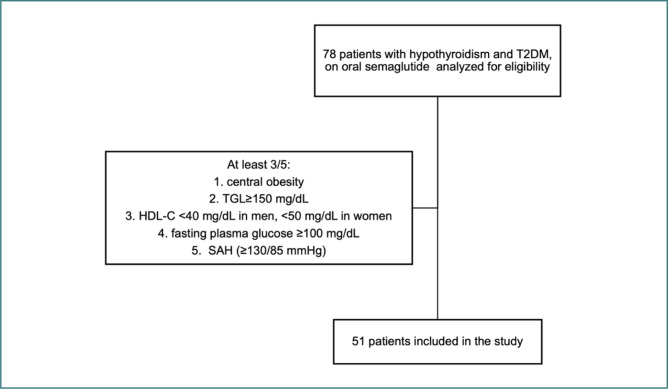
Evaluation of patients for eligibility. TGL, triglycerides; HDL-C, High-Density Lipoprotein Cholesterol; SAH, Systemic Arterial Hypertension.

The purpose of this retrospective investigation was to evaluate how oral semaglutide influences key MetS components in a cohort of patients with coexisting T2DM and hypothyroidism. By integrating endocrinological and cardiometabolic perspectives, this study aims to clarify the therapeutic potential of semaglutide in a complex and frequently overlapping pathophysiological setting.

## MATERIAL AND METHODS

This retrospective cohort study aimed to evaluate the impact of semaglutide treatment over 6 months in patients with hypothyroidism and T2DM, focusing on key metabolic markers: body mass index (BMI), HbA1c, triglycerides, HDL-C, and LDL-C. A database of 78 patients who presented to the Prof. Dr. N. C. Paulescu National Institute of Diabetes, Nutrition and Metabolic Diseases was reviewed.

The study population consisted of patients diagnosed with (1) hypothyroidism (of either autoimmune or post-procedural etiology), (2) T2DM, and who also fulfilled the diagnostic criteria for (3) metabolic syndrome, defined by the presence of at least three of the following: a. central obesity (BMI ≥30kg/m2), b. hypertriglyceridemia (≥150 mg/dL), c. decreased serum high-density lipoprotein cholesterol (HDL-C <40 mg/dL in men, <50 mg/dL in women), d. elevated fasting plasma glucose (≥100 mg/dL), e. systemic arterial hypertension (≥130/85 mmHg). Based on these criteria, 51 patients were included in the final analysis, while the remaining 27 did not meet the diagnostic threshold for MetS (Figure 1).

Given that we were concurrently conducting another study investigating the effects of semaglutide on thyroid function, we also compared baseline and 6-month post-treatment thyroid-stimulating hormone (TSH) levels in the patients included in the current study.

The database was compiled using Microsoft Excel. Dynamic values of the metabolic parameters were analyzed at baseline and after 6 months of treatment with oral semaglutide (14 mg daily). These data were processed using appropriate statistical tests (paired-sample *t*-tests), along with effect size measurement using Cohen’s d. Once the data were collected, JASP Software (version 0.19.3, JASP Team, Amsterdam, The Netherlands) was used for statistical analysis.

An a priori power analysis was performed to estimate the minimum sample size required for detecting a moderate within-subject effect (Cohen’s d = 0.5) with 80% statistical power at a two-tailed α = 0.05, using a paired *t*-test.

## RESULTS

According to the inclusion criteria, 51 of the 78 identified patients were included in the study. Among them, approximately 88% were women (*n* = 45), 43% were over 65, and the rest were aged 45-64. At baseline, 47 of the included patients had dyslipidemia (92%), and among them, over 50% met the diagnostic criteria for metabolic syndrome. Forty were diagnosed with arterial hypertension, which represents 78% of the patients included in the study. Over the 6 months, the blood pressure values among these patients remained largely stable, with only minor fluctuations. Accordingly, no substantial or statistically significant changes were detected. Moreover, at the time of initial presentation, almost 50% of patients could be classified as having obesity (any class). This percentage had decreased to approximately 37% after 6 months. Table 1 summarizes these data.

The values of key metabolic markers at baseline and after 6 months were compared to assess the effect of semaglutide treatment on these parameters. At the initial measurement, the mean BMI was 31.5 kg/m2 ± 4.8 SD, which decreased to 30.1 kg/m2 ± 4.3 SD 6 months later (*P* < 0.001). An average reduction in BMI of 1.5 kg/m2 (4.04%) was observed over the 6 months. Additionally, an improvement in T2DM control was noted: mean HbA1c at the 6-month evaluation was 7.5% ± 0.7 SD, compared to an initial mean HbA1c of 8.02% ± 0.8 SD (*P* < 0.001). This represents an absolute reduction of 0.52 percentage points (6.7%). The lipid profile also changed over time, indicating a reduction in the severity of the associated cardiovascular risk. The levels of LDL-C decreased significantly over the 6 months. The baseline LDL-C value was 128.3 mg/dl ± 34 SD, which decreased to 113.6 mg/dl ± 28 SD (*P* < 0.001). This represents an absolute reduction of 14.7 mg/dl (a mean relative decrease of approximately 11.6%). Consistently, triglycerides (TG) also declined, from 168.9 mg/dL ± 66.3 SD at baseline to 153.8 mg/dL ± 48.1 SD (*P* < 0.001), representing a mean decrease of 15.1 mg/dL (6.7%). Conversely, HDL-C increased by 2.7 mg/dL (9%), rising from 43.8 mg/dL ± 10.7 SD to 46.5 mg/dL ± 8.8 SD after 6 months (*P* = 0.002). All these data, both the values and their statistical significance, are summarized in Table 2.

**Table 1:** Demographic and clinical characteristics of patients

Characteristics of patients	*n* (%)	
**Age (years)**		
≥ 65	22 (43.1%)	
45-64	29 (56.9)	
**Sex**		
Female	45 (88%)	
Male	6 (12%)	
**Hypertension**	40 (78%)	
**BMI**	**First presentation**	**Six months later**
Class III obesity	2 (3.9%)	2 (3.9%)
Class II obesity	8 (15.6%)	2 (3.9%)
Class I obesity	17 (33.3%)	15 (29.4%)
Overweight	22 (43.1%)	28 (54.9%)
**TGL≥150 mg/dl**	26 (50.9%)	21 (41.1%)
**HDL <40 mg/dl in males**	2 (3.9%)	2 (3.9%)
**HDL<50 mg/dl in females**	27 (52.9%)	25 (49%)

BMI, Body Mass Index; Overweight: BMI = 25.0–29.9 kg/m^2^; Class I obesity: BMI = 30.0–34.9 kg/m^2^; Class II obesity: BMI = 35.0–39.9 kg/m^2^; Class III obesity: BMI ≥ 40.0 kg/m^2^; TG, Triglycerides; HDL-C, High-Density Lipoprotein Cholesterol

As previously mentioned in the Material and Methods section, although thyroid function was not the primary focus of this study, we evaluated TSH levels and observed a mean decrease of 0.7% from baseline to the 6-month follow-up. At baseline, the mean TSH value was 4.2 μIU/mL ± 3.7 SD, and after 6 months, it decreased to an average of 2.9 μIU/mL ± 1.8 SD. Thyroid function has a significant impact on the progression of metabolic syndrome, making it a particularly important aspect. While this aspect was not within the scope of our current study, a forthcoming study will conduct a comprehensive analysis of the relationship between thyroid hormone dynamics and semaglutide treatment.

**Table 2:** Changes in metabolic parameters

	First presentation	6 months later	*P* value	Cohen’s d
BMI (kg/m^2^)	31.5 ± 4.8	30.1 ± 4.3	<0.001	1.1
HbA1c (%)	8.02 ± 0,8	7.5 ± 0,7	<0.001	1.5
LDL-C (mg/dl)	128.3 ± 34	113.6 ± 28	<0.001	0.73
TG (mg/dl)	168.9 ± 66.3	153.8 ± 48.1	<0.001	0.52
HDL-C (mg/dl)	43.8 ± 10.7	46.5 ± 8.8	0.002	0.43

## DISCUSSION

Our retrospective cohort study demonstrates that oral semaglutide improved multiple components of the MetS—including reductions in HbA1c, BMI, triglycerides, and LDL-cholesterol, along with increases in HDL-cholesterol—in patients with coexisting hypothyroidism and T2DM. These findings align with outcomes reported in the broader T2DM population treated with oral semaglutide in the PIONEER trial program, which similarly documented improvements in glycemic control, body weight, and cardiometabolic risk factors [24-28].

In our cohort of patients with type 2 diabetes and hypothyroidism treated with oral semaglutide (14 mg) and levothyroxine (mean dose of 75 mcg/day), we observed a 4% reduction in BMI and significant improvements in lipid profile over 6 months. When compared with the PIONEER trials, which reported HbA1c reductions of approximately 1.2–1.4% and weight loss of 1–3% with semaglutide alone, our findings suggest comparable glycemic efficacy and a slightly greater impact on weight parameters [25].

In contrast to PIONEER 1, which primarily demonstrated significant reductions in HbA1c and modest decreases in body weight (approximately 1–3%) without reporting detailed lipid outcomes, our six-month cohort of patients with coexisting hypothyroidism and type 2 diabetes receiving oral semaglutide (14 mg) plus levothyroxine showed a broader metabolic benefit; we observed marked improvements in triglycerides (−6.7%), increases in HDL cholesterol (+9%), and favorable trends in LDL cholesterol, indicating a more comprehensive impact on cardiometabolic risk factors.

While our findings indicate concurrent improvements in weight and lipid parameters among patients receiving both oral semaglutide and levothyroxine, the observational nature of this analysis precludes any conclusions regarding causality or drug interaction. Thyroid hormone replacement may influence metabolic responses to GLP-1 receptor agonist therapy; however, this remains speculative and cannot be confirmed without an appropriate control group not receiving levothyroxine. The observed trends should therefore be interpreted as hypothesis-generating, highlighting an area that merits further investigation. Prospective, controlled studies are needed to determine whether concomitant thyroid hormone replacement therapy modifies the metabolic effects of semaglutide in a consistent and clinically meaningful manner.

From a pharmacodynamic perspective, several mechanisms may plausibly contribute to the metabolic improvements observed in our cohort. Oral semaglutide has been shown to delay gastric emptying and to enhance both fasting and postprandial glucose and lipid metabolism in patients with T2DM [29,30]. These physiological effects may, at least in part, explain the reductions in HbA1c, BMI, and serum lipid concentrations noted in our analysis. Nevertheless, given the observational design, these associations should be interpreted as supportive rather than confirmatory evidence of drug-mediated mechanisms.

The interplay between hypothyroidism and MetS is well-established, with subclinical hypothyroidism (SCH) being associated with an approximately 2.5-fold higher risk of MetS [9]. Hypothyroidism can exacerbate insulin resistance, dyslipidemia, and visceral adiposity, thereby amplifying cardiometabolic burden. Within this context, the metabolic responses observed with oral semaglutide in patients receiving concomitant levothyroxine may reflect a favorable interaction between improved thyroid status and GLP-1 receptor–mediated pathways, rather than a direct synergistic effect. Our findings thus contribute preliminary, hypothesis-generating insights into this complex metabolic phenotype, underscoring the need for prospective studies to delineate the distinct and combined roles of GLP-1 receptor agonism and thyroid hormone replacement in metabolic regulation.

Another potential benefit concerns thyroid function itself. A recent investigation noted that patients with hypothyroidism who lost weight on GLP-1 receptor agonists—specifically on semaglutide—experienced reductions in TSH levels (in the order of −0.55 mU/L for semaglutide)[31,32]. Although our study did not include longitudinal assessment of TSH dynamics, the findings suggest that semaglutide may exert beneficial effects beyond classical metabolic outcomes, potentially including modulation of the thyroid axis.

However, caution is reasonable. There have been some isolated case reports suggesting that semaglutide (particularly in its intravenous form) may influence thyroid function negatively, potentially inducing subclinical hypothyroidism, which resolves after discontinuation of therapy [33]. Such conflicting observations underscore the importance of vigilant thyroid function monitoring when prescribing GLP-1 receptor agonists to hypothyroid patients, especially given pharmacokinetic interactions—specifically delayed gastric emptying—that may reduce levothyroxine absorption [34,35].

To date, evidence does not support a clinically meaningful interaction between oral semaglutide and levothyroxine. In a pharmacokinetic study of healthy volunteers, oral semaglutide (14 mg) modestly increased total T_4_ exposure. However, it did not alter peak levels or require dose adjustments. Any interaction appears to be pharmacokinetic and limited [36]. Our observations should therefore be viewed as exploratory.

Future research from our group will focus on longitudinal assessment of thyroid parameters, including TSH, free T_4_, and free T_3_, to identify reproducible response patterns and confirm these findings in an expanded cohort.

Despite the points above, semaglutide remains compelling: it improves glycemic control, supports weight loss, reduces adverse cardiovascular-renal outcomes in T2DM, and may benefit lipid metabolism [25, 37–39].

### Limitations and future directions

Our study’s retrospective, single-center design and relatively small sample size (*n* = 51) highlight the importance of future prospective, randomized trials featuring larger cohorts. In our upcoming publication, we intend to expand on these findings by comparing two well-defined groups: patients receiving only oral semaglutide versus those receiving levothyroxine sodium alone. This exploratory analysis will serve as the foundation for future expanded studies. Additionally, we plan to incorporate systematic thyroid function assessments, including TSH and free T_4_/T_3_.

An important consideration in interpreting our findings is the marked gender imbalance within the study population, with women representing 88% of the included patients. This disproportion reflects, to a large extent, the epidemiological characteristics of the source population rather than a selection bias. Hypothyroidism is significantly more prevalent among women, with a female-to-male ratio estimated at 5–8:1 in most cohorts, largely attributed to autoimmune thyroiditis as the leading etiology. Consequently, the predominance of female participants in our cohort mirrors the real-world distribution of hypothyroidism in patients with T2DM and MetS. Future research should aim to include more balanced cohorts to determine whether sex-related differences influence the therapeutic efficacy or metabolic outcomes of oral semaglutide in this dual-pathology population.

The anthropometric measure chosen is another drawback of the current study: we used BMI rather than waist circumference to assess adiposity. Because BMI is readily accessible and frequently documented in clinical practice, it may occasionally be unable to accurately identify central adiposity, a crucial aspect of MetS, due to its inability to differentiate fat distribution. In fact, much data has demonstrated that, particularly among BMI groups, waist circumference is frequently a better indicator of cardiometabolic risk, including morbidity and mortality [40]. Nonetheless, BMI and waist circumference often show comparable predictive value for diabetes risk in large populations—for example, in a retrospective longitudinal cohort, both measures performed similarly in forecasting the onset of diabetes [41]. Our decision to use BMI is grounded in its widespread application in oral semaglutide trials, which informed our design and serve as methodological precedents. Within the broader PIONEER clinical programs—investigating oral semaglutide at various doses in people with type 2 diabetes—baseline BMI was used as a stratification variable to assess differential responses in HbA1c and body weight outcomes [26–28]. These studies consistently monitored BMI dynamics throughout treatment, also providing meaningful context for its use in our exploratory analysis.

In addition, we acknowledge, as a limitation, the absence of detailed information on concomitant treatments, which may have introduced unmeasured bias. Nevertheless, all concomitant medications, including statins, antihypertensive agents, and other antidiabetic therapies, were reported to have remained stable throughout the observation period. No new treatments were introduced, and no changes in statin dosage were made. This potential source of uncertainty was duly considered in the interpretation of our findings.

Finally, the assumed baseline predictors of response— such as BMI and TSH levels in our study—suggest that personalized medicine approaches could optimize semaglutide use in this high-risk subgroup. Future research should aim to stratify patients based on these and other biomarkers to guide therapeutic decisions more precisely.

## CONCLUSION

In this retrospective observational study, oral semaglutide was associated with improvements across many components of MetS—including glycemic control, adiposity, and lipid parameters—in individuals with coexisting hypothyroidism and type 2 diabetes mellitus. While these associations suggest potential metabolic benefits within this complex, high-risk phenotype, the findings should be interpreted with caution, as the study design does not allow for causal inference.

Patients diagnosed with hypothyroidism who are presenting with higher baseline BMIappeared to show more notable metabolic responses, suggesting that baseline metabolic and endocrine characteristics may influence treatment outcomes. This observation underscores the potential importance of baseline stratification for optimizing therapeutic decisions in individuals with concurrent endocrine and metabolic dysfunction.

While these results are promising, they should be considered hypothesis-generating. Prospective, adequately powered clinical trials that include comprehensive thyroid profile assessment and long-term cardiovascular endpoints are necessary to confirm these associations and to more precisely define the efficacy, safety, and durability of semaglutide therapy in populations with dual pathology.

Collectively, our results add to the growing evidence indicating that GLP-1 receptor agonist therapy may play a supportive role in the personalized management of patients with coexisting thyroid and metabolic dysfunctions, while emphasizing the importance of careful interpretation and thorough future validation.
